# Pinpointing secondary metabolites that shape the composition and function of the plant microbiome

**DOI:** 10.1093/jxb/eraa424

**Published:** 2020-09-30

**Authors:** Richard P Jacoby, Anna Koprivova, Stanislav Kopriva

**Affiliations:** 1 Institute for Plant Sciences, Cluster of Excellence on Plant Sciences (CEPLAS), University of Cologne, Cologne, Germany; 2 The James Hutton Institute, UK

**Keywords:** Benzoxazinoids, camalexin, coumarins, glucosinolates, microbial community assembly, plant–microbe interactions, secondary metabolites, triterpenes

## Abstract

One of the major questions in contemporary plant science involves determining the functional mechanisms that plants use to shape their microbiome. Plants produce a plethora of chemically diverse secondary metabolites, many of which exert bioactive effects on microorganisms. Several recent publications have unequivocally shown that plant secondary metabolites affect microbiome composition and function. These studies have pinpointed that the microbiome can be influenced by a diverse set of molecules, including: coumarins, glucosinolates, benzoxazinoids, camalexin, and triterpenes. In this review, we summarize the role of secondary metabolites in shaping the plant microbiome, highlighting recent literature. A body of knowledge is now emerging that links specific plant metabolites with distinct microbial responses, mediated via defined biochemical mechanisms. There is significant potential to boost agricultural sustainability via the targeted enhancement of beneficial microbial traits, and here we argue that the newly discovered links between root chemistry and microbiome composition could provide a new set of tools for rationally manipulating the plant microbiome.

## Teaser

We provide an update of the specific plant secondary metabolites that shape the microbiome, emphasizing newly discovered links between root chemistry and microbiome composition.

## Introduction

Historically, the vast majority of the literature on plant–microbe interactions studied a small set of nutrient-mobilizing symbionts and agricultural pathogens (Glazebrook *et al.*, 1997; Udvardi and Poole, 2013). However, there has been a conceptual shift during the last 20 years, with new sequencing technologies revealing that all non-sterile plants are colonized by a diverse microbiome, which exerts a strong influence on plant productivity in both natural and agricultural settings (Bulgarelli *et al.*, 2013; Müller *et al.*, 2016). Akin to medical science, plant microbiome research has rapidly emerged as a major scientific frontier, because it is increasingly appreciated that microbial science will play a growing role in future agricultural systems (Finkel *et al.*, 2017).

### Functional significance of the microbiome for plant and crop performance

Metagenomics surveys show that the number of enzymatic functions encoded in the microbiome vastly outweighs the enzymatic capability of the plant itself (Busby *et al.*, 2017). Some of the functional activities undertaken by the microbiome can directly benefit plant performance, with most attention being paid to three distinct mechanisms: (i) the improvement of plant nutrition (Jacoby *et al.*, 2017); (ii) the suppression of pathogen outbreaks (Pieterse *et al.*, 2014); or (iii) the modulation of abiotic stress tolerance (Cheng *et al.*, 2019). In agriculture, the targeted enhancement of these desirable microbial activities offers a potential avenue towards maintaining crop yields while reducing the application of synthetic fertilizers and pesticides (Bender *et al.*, 2016).

Although there is significant potential to boost agricultural sustainability by incorporating microbiome science into farm practice, there are still major knowledge gaps that restrict our capacity to rationally manipulate the plant microbiome (Tosi *et al.*, 2020). For instance, one option to promote desirable plant–microbe interactions involves selecting crop varieties according to their ability to recruit beneficial microbes into the rhizosphere. However, this strategy is not yet commercially feasible due to an incomplete knowledge of how plant genetics affects the microbiome, as well as a lack of high-throughput screening tools for phenotyping microbial-mediated traits in large breeding populations (Cordovez *et al.*, 2019; Wille *et al.*, 2019). Another option involves inoculating seeds or crops with beneficial microbes. Although a wide range of microbial crop inoculants are commercially available, their uptake by farmers is relatively limited, with the exception of N_2_-fixing *Rhizobium* inocula that are routinely applied onto legumes (O’Callaghan, 2016). One of the criticisms of crop inoculants is that they often deliver unpredictable results in field settings, frequently because the inoculated strains are poorly adapted to local soil conditions, or because they fail to colonize the host plant in competition against environmental strains (Kaminsky *et al.*, 2019). Moreover, there is still significant uncertainty surrounding what constitutes a ‘healthy’ or ‘beneficial’ microbiome (Trivedi *et al.*, 2020). In order to improve the efficacy of microbiome-based amendments in agriculture, a future research priority involves unravelling how final crop yield is influenced by the complex plant genotype×microbiome×environment×management interaction (Busby *et al.*, 2017).

### Dissecting the factors shaping microbiome assembly

Over the last 20 years, a plethora of studies have quantified the factors that influence plant microbiome composition. Integrating these results together, it is evident that soil is the main source of inoculum and therefore exerts the strongest effect upon microbiome composition, whereas the host genotype fine-tunes what the soil provides the plant with (Bulgarelli *et al.*, 2012; Lundberg *et al.*, 2012). Although this plant genotype effect is relatively weak, it is often linked to specific microbial taxa which can dramatically modulate host fitness. Mechanistically, microbiome assembly can be modulated by multiple plant traits, such as immune responses, root morphology, and metabolite composition (Rodriguez *et al.*, 2019). Each of these traits offers a potential target for crop breeding strategies aiming to recruit desirable microbial strains, and this review will focus on the role played by secondary metabolites.

## Metabolic interdependence between plants and microbes

When considering plant–microbe interactions from a metabolic perspective, it is well understood that plants fuel the proliferation of microbial life in the rhizosphere by depositing carbon substrates below-ground (Sasse *et al.*, 2018). These rhizodeposits account for ~10% of the plant’s carbon budget (Pausch and Kuzyakov, 2018), and the microbial utilization of plant-derived growth substrates explains why the rhizosphere contains a dramatically higher abundance of microbes compared with bulk soil (Rovira, 1965). The microbial strains recruited to the rhizosphere can exert a spectrum of effects upon plant performance, ranging from pathogenic to mutualistic. Several adaptive plant phenotypes are actually mediated by microbial associations, such as nutrient uptake, pathogen suppression, and stress tolerance (Mendes *et al.*, 2011; Haney *et al.*, 2015; Castrillo *et al.*, 2017). Over evolutionary time, it is postulated that the ecological success of plants was dependent upon their ability to recruit cooperative strains to the rhizosphere (Vandenkoornhuyse *et al.*, 2015). Therefore, scientific research that defines the metabolic mechanisms used by plants to recruit beneficial microbes could provide a new set of breeding targets for crop improvement (Wei and Jousset, 2017).

A substantial field of literature has investigated the specific molecules that are exchanged between plants and microbes (Sasse *et al.*, 2018; Stringlis *et al.*, 2018; Cotton *et al.*, 2019; Huang *et al.*, 2019; Jacoby *et al.*, 2020). Historically, the overwhelming focus of these studies involved documenting the microbial consumption of the primary metabolites contained in root exudates, particularly sugars, amino acids, and organic acids (Canarini *et al.*, 2019). This focus is supported by a solid physiological basis, because these abundant primary metabolites are loaded into the phloem, transported to the root, and then exuded at the root apical meristem, which is the major site of below-ground sugar deposition (Farrar *et al.*, 2003). Once released into the soil, primary metabolites serve as labile growth substrates that are rapidly consumed by fast-growing generalist microbial strains (Goldfarb *et al.*, 2011). However, the microbial consumption of primary metabolites does not provide a comprehensive explanation accounting for microbial community assembly, because recent investigations of rhizosphere microbiome composition have revealed that a huge diversity of taxa assemble on and around plant roots, including a relatively high proportion of slow-growing strains adapted to specialized metabolic niches (Zhalnina *et al.*, 2018). This suggests that the metabolic interplay between plants and microbes involves a wider spectrum of metabolites, comprising both primary and secondary metabolites.

Physiologically, there are two major reasons why rhizosphere microbes would have access to a wide diversity of plant-derived secondary metabolites. First, many rhizosphere microbial strains are actually endophytes that colonize internal spaces within the root (Gaiero *et al.*, 2013), where they would have access to the full chemical diversity of living plant cells. Secondly, a significant amount of rhizodeposition occurs via the lysis of sloughed-off root cells (Dennis *et al.*, 2010), and these lysates would presumably contain a diverse mixture of metabolic classes. Both of these mechanisms imply that plant secondary metabolites would play a prominent role in shaping microbial community assembly.

## Evolution and ecology of plant secondary metabolites

Secondary metabolites are broadly defined as molecules that are not essential to organismal growth and development, which differentiates them from primary metabolites (Wink, 2003; Kliebenstein and Osbourn, 2012). Compared with other organisms, plants produce a rich and diverse array of secondary metabolites. Many of these molecules exert pharmacological activity, which explains why drug discovery pipelines have a long history of researching bioactive plant natural products (Schmidt *et al.*, 2008). Biochemically, secondary metabolites derive from precursors in primary metabolism, whereby the molecular structure of the precursor is usually modified via the successive action of specialized enzymes. Many of these enzymes appear to have resulted from gene duplication events, whereby an essential gene from primary metabolism was duplicated, providing a redundant copy that could subsequently evolve a new function under relaxed selection pressure (Ober, 2005). Gene duplication is extensive amongst the angiosperms, and this massive expansion of the genetic repertoire occurring in higher plant evolution seems to be a central factor contributing to the tremendous diversification of secondary metabolite profiles across plant species adapted to various ecological niches (De Bodt *et al.*, 2005).

Plant secondary metabolites were widely considered as metabolic waste products until roughly the 1970s, when the emergence of chemical ecology as a scientific discipline enabled researchers to study how secondary metabolites were a central mechanism mediating the interactions between plants and other organisms (Hartmann, 2007). This includes cooperative interactions, such as plants recruiting insect pollinators via volatile emissions, and also antagonistic interactions, such as plants deterring herbivores via production of unpalatable or toxic metabolites. Generally, the majority of the early chemical ecology literature focused on plant–animal interactions rather than plant–microbe interactions. Amongst those studies that did focus on microbes, most of the literature investigated how plant secondary metabolites can modulate the interactions with individual microbial strains, particularly pathogens and symbionts (Table 1). However, this situation is now changing, because new methodological tools developed for microbiome-scale studies have recently been applied to study how secondary metabolites affect the composition and function of entire microbial communities.

**Table 1.** Overview of chemical structures, metabolic precursors, and microbial effects for secondary metabolites that shape the root microbiome

Secondary metabolite	Example chemical structure	Precursor primary metabolite	Mechanistic action on microbes
Benzoxazinoids	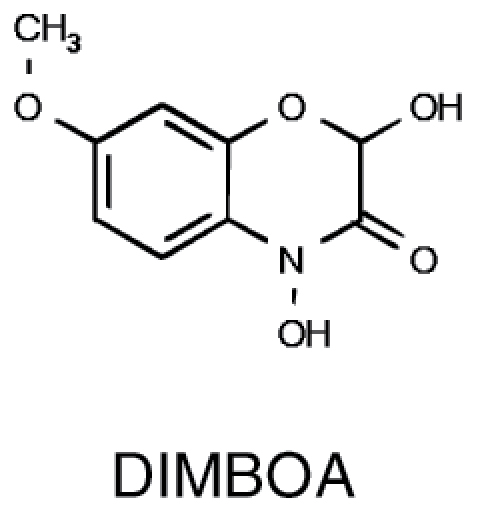	• Chorismate	• Chemoattractant (Neal *et al.*, 2012*a*) • Modification of -SH and -NH_2_ groups in proteins, leading to enzyme inactivation (Wouters *et al.*, 2016)
Camalexin	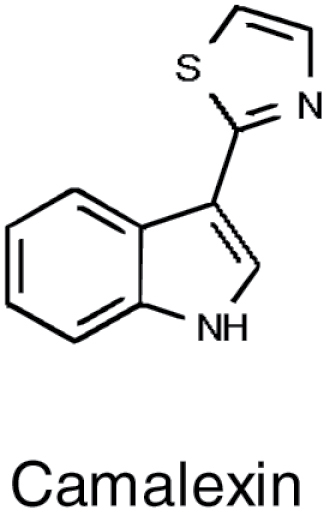	• Tryptophan	• Disruption of membrane integrity (Rogers *et al.*, 1996)
Coumarins	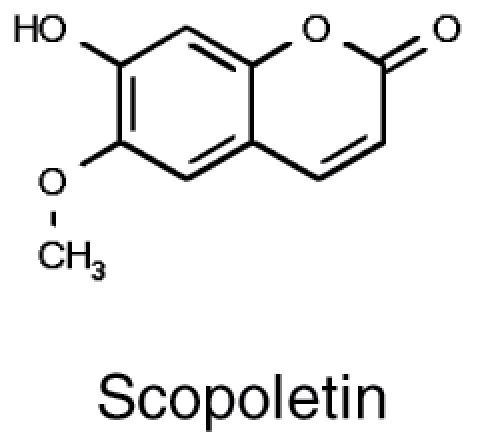	• Phenylalanine	• Disruption of transcription (Yang *et al.*, 2016) • Disruption of quorum sensing and biofilm formation (Yang *et al.*, 2016) • Damage to membranes (Yang *et al.*, 2016)
Flavonoids	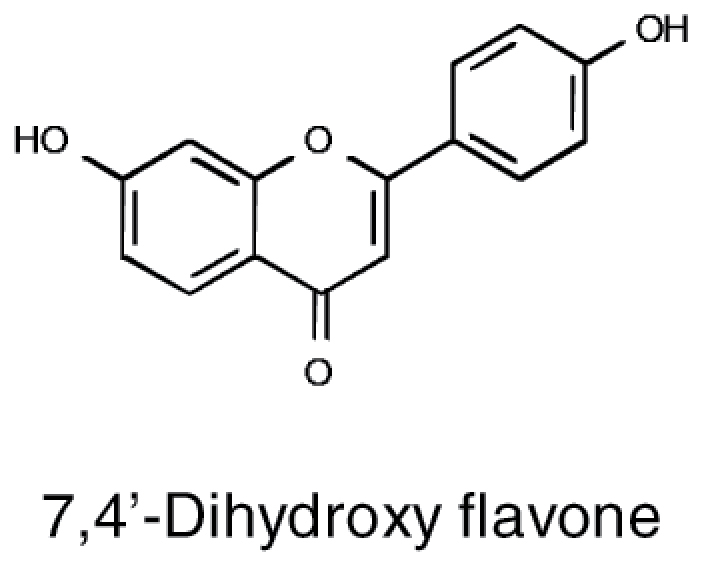	• Phenylalanine	• Induction of *nod* gene expression in *Rhizobium* (Redmond *et al.*, 1986) • Damage to membranes (Tsuchiya and Iinuma, 2000) • Inhibition of enzymes (Zhang and Rock, 2004) • Disruption of nucleic acid synthesis (Mori *et al.*, 1987) • Disruption of biofilm formation (Vikram *et al.*, 2010)
Glucosinolates	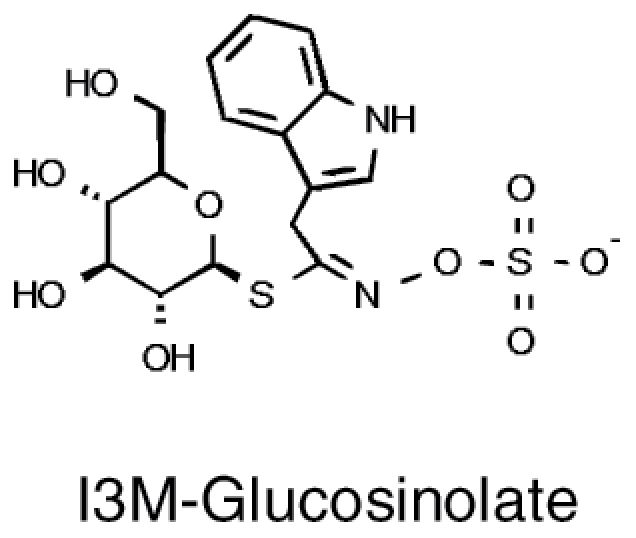	• Tryptophan • Phenylalanine • Methionine	• Isothiocyanate-mediated enzyme inactivation (Aires *et al.*, 2009) • Isothiocyanate-mediated disruption of membrane integrity (Borges *et al.*, 2015)
Strigolactones	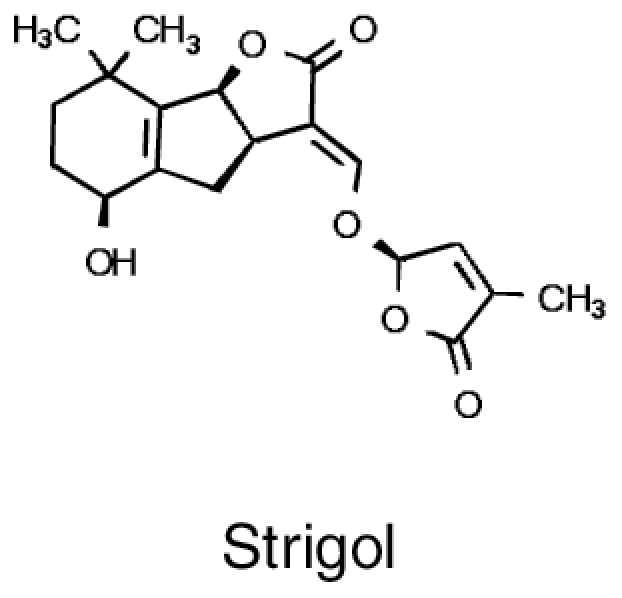	• Isopentenyl pyrophosphate • β-Carotene	• Induction of hyphal branching in mycorrhizal fungi (Akiyama *et al.*, 2005)
Triterpenes	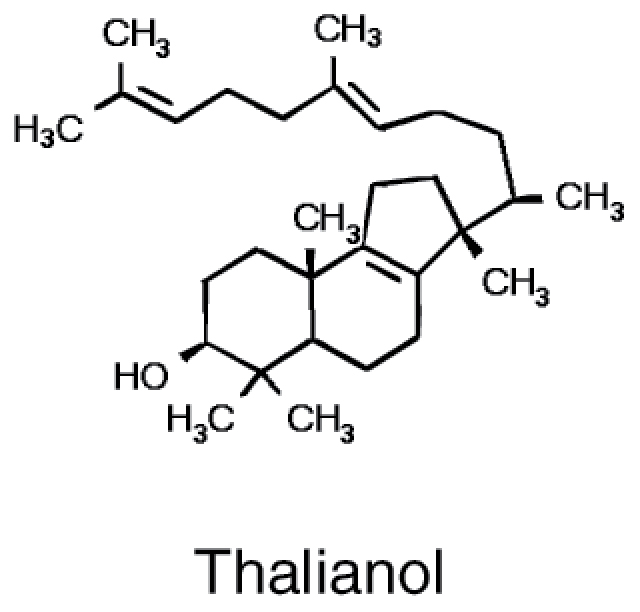	• Isopentenyl pyrophosphate • Squalene	• Disruption of membrane integrity (de León *et al.*, 2010)

## New tools and approaches to characterize specific metabolites that shape the microbiome

**Fig. 1.**
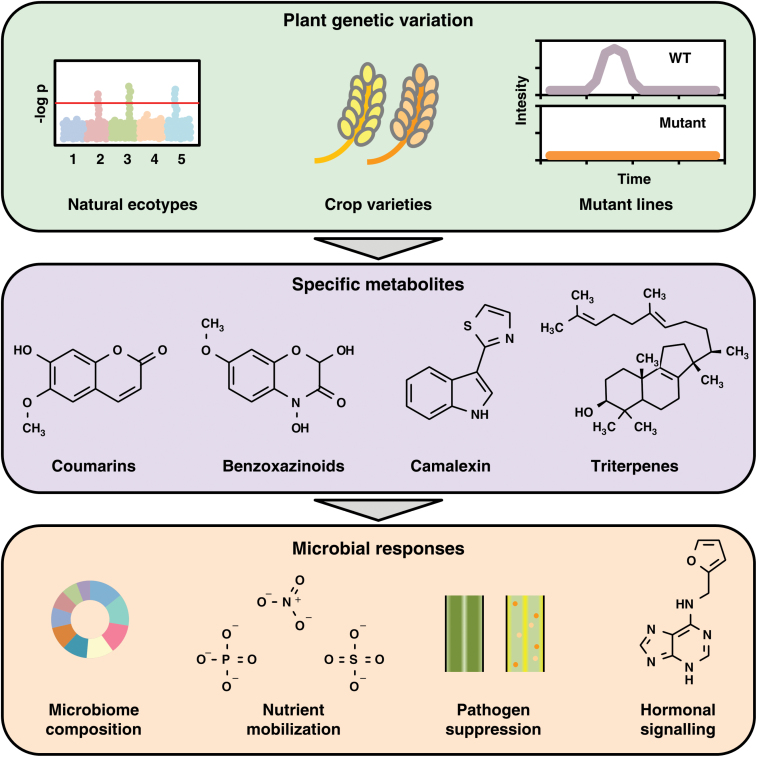
Secondary metabolites are a mechanistic link between plant genetics and microbiome functions. The top panel shows genetic tools for generating plants with altered secondary metabolite profiles. The middle panel illustrates four secondary metabolites recently shown to affect microbiome composition and function. The bottom panel shows microbial responses elicited by variations in secondary metabolite abundance.

For several decades, it has been known that legumes secrete flavonoid secondary metabolites as a mechanism to recruit nitrogen-fixing Rhizobia symbionts (Redmond *et al.*, 1986). In recent years, a suite of new publications has characterized how specific secondary metabolites can shape the root microbiome of Arabidopsis and maize, by modulating the abundance or function of distinct microbial strains (Fig. 1) (Lebeis *et al.*, 2015; Stringlis *et al.*, 2018; Cotton *et al.*, 2019; Huang *et al.*, 2019; Koprivova *et al.*, 2019; Voges *et al.*, 2019). Methodologically, these new studies have utilized a set of emerging tools and approaches for investigating plant–microbe interactions. Specifically, this includes screening microbial responses across diverse panels of plant genotypes using panels of natural accessions or mutants in specific metabolic pathways (Stringlis *et al.*, 2018; Koprivova *et al.*, 2019). On the microbial side, natural soil or defined synthetic communities (SynComs) have been successfully used. In particular, the availability of microbial collections offers great opportunities to generate microbial SynComs with a defined and controlled diversity to address various research questions (Bai *et al.*, 2015; Vorholt *et al.*, 2017).

### Synthetic communities as simplification of complex microbial assemblies

Most of the reports use SynComs that represent the taxonomic assembly of the microbiota (Bodenhausen *et al.*, 2014; Lebeis *et al.*, 2015; Niu *et al.*, 2017). This enables a simplified analysis of the effects of plant genotypes on the microbial assembly. Using a community of the seven most abundant taxa, Bodenhausen *et al.* (2014) revealed that changes in cuticular properties have a large impact on the associated bacteria in the leaves. Interestingly, the effects were different for different taxa, showing the ability of plants to shape not just the overall quantity of associated microbes, but also the quality (i.e. the composition of the communities). This has been confirmed by analyses of several accessions, which also showed a different impact on the growth of the SynComs (Bodenhausen *et al.*, 2014). A more complex SynCom of 38 strains isolated from Arabidopsis roots was used to dissect the contribution of plant hormones to root microbiome assembly (Lebeis *et al.*, 2015). Analysis of a panel of phytohormone mutants showed the importance of salicylic acid for exclusion of certain bacterial strains from colonizing the root. This was confirmed by treatment with exogenous salicylate, showing that, indeed, metabolites in the rhizosphere affect the ability of microbes to interact with the plant host (Lebeis *et al.*, 2015). A similar SynCom with 35 members was instrumental in finding a coordination between phosphate starvation response, immunity, and microbiome assembly, and the identification of PHOSPHATE STARVATION RESPONSE 1 (PHR1) as the key regulator of this integration (Castrillo *et al.*, 2017). A Syncom based on a similar principle but reduced to 22 members helped to dissect a role for coumarins in shaping the root microbiome (Voges *et al.*, 2019).

The SynComs used in these and other studies were chosen to represent a simplified assembly of strains based on the taxonomical composition of the full microbiome. Across the literature, the SynComs used differ in their size, from small communities of ~7 members (Bodenhausen *et al.*, 2014) to multi-kingdom communities of >200 strains (Durán *et al.*, 2018). So what is the optimal SynCom size? While greater size of the SynComs is a better proxy for the functional diversity of the endogenous plant microbiome, it may also affect the stability of the community. This has been rarely explicitly tested, and to date probably only one SynCom can reliably be called stable, based on rigorous testing (Niu *et al.*, 2017). The maize root SynCom is composed of seven strains representing all the major phyla, which were obtained through host-driven selection from more complex inocula (Niu *et al.*, 2017). The community is stable and develops consistently in independent replicates, but only when complete. Removals of single strains lead to changes in the composition, and removal of one particular strain, *Enterobacter cloacae*, leads to a complete domination of another strain, *Curtobacterium pusillum*. One limitation of SynComs is that they only grasp a small fraction of the functional traits that would be contained in a natural microbiome, and are therefore poorly suited to unbiased discovery of microbiome functions. However, the work of Niu *et al.* (2017) shows the power of SynComs for identifying putative microbial hubs that govern community assembly, thus demonstrating that SynComs are an excellent resource to study the mechanisms that underlie stability of bacterial communities.

### Exometabolomics

Such studies will be feasible due to recent developments in exometabolomics (Erbilgin *et al.*, 2017; Jacoby and Kopriva, 2019). Exometabolomics, also called metabolic footprinting, uses the methods of metabolic analysis—LC-MS, GS-MS, or NMR—to analyse extracellular metabolites (Allen *et al.*, 2003). It is a simplified way to provide information about metabolic effects of gene mutations in microorganisms or their communication mechanisms, and has been successfully used to support metabolic engineering and industrial biotechnological processes (Allen *et al.*, 2003; Mapelli *et al.*, 2008; Zha *et al.*, 2014). In the context of microbiome research, exometabolomics can be used to identify metabolites used for cross-feeding among strains in a SynCom in a similar way to how it was used to identify strains capable of complementation of auxotroph mutants in a biotechnological setting (Kosina *et al.*, 2016). Exometabolomics in combination with growth assays on spent media is a powerful approach to identify metabolites enabling or inhibiting growth and so dissect the dynamics of the microbial communities. To move this approach towards better fit for plant microbe research, root extracts or exudates have been successfully used as the carbon source for the microbes (Jacoby *et al.*, 2018; Zhalnina *et al.*, 2018). In experiments using Arabidopsis root extracts, Jacoby *et al.* (2018) showed that root-associated bacteria are capable of using a much more diverse spectrum of metabolites than *Escherichia coli*, and that the individual strains also widely differ in this capability. Zhalnina *et al.* (2018) used root exudates from *Avena barbata* to reveal that changes in exudate composition during plant development together with bacterial preferences for uptake of certain metabolites, such as organic acids, determine root microbiome assembly. These reports show the power of this approach and the directions in which it could be developed to dissect the metabolic interdependencies in the plant microbiome, both between individual strains and between the host and the microbiota. In particular, the analyses using microbes growing on exudates or extracts from plant mutants, which display differences in microbiome assembly and/or function, have the potential to discover the key metabolites shaping the communities.

### Natural variation and genome-wide association studies

The mutants are just one part of genetic resources that can be used to disentangle the metabolic dependencies in plant–microbe interactions. Their power was clearly demonstrated in the identification of several metabolites important for microbiome assembly, such as salicylic acid, coumarins, and other secondary metabolites (Lebeis *et al.*, 2015; Stringlis *et al.*, 2018; and see below). However, to use mutant collections requires previous knowledge and is biased towards investigations of rather obvious candidate signals. In contrast, using natural variation for genome-wide association studies (GWAS) allows identification of the mechanisms in an unbiased manner and possibly can uncover unexpected links (Weigel, 2012). Microbiome composition differs not only between species but also between accessions/varieties of the same species, such as Arabidopsis or maize (Micallef *et al.*, 2009; Bulgarelli *et al.*, 2012; Lundberg *et al.*, 2012; Peiffer *et al.*, 2013). In addition, clear heritable differences in root exudate composition have been determined among Arabidopsis accessions (Mönchgesang *et al.*, 2016). Thus, as plant microbiomes are shaped by metabolites in the rhizosphere, it is feasible to use microbiota traits as phenotypes to identify plant genes through GWAS.

A number of GWAS have addressed various immunity-related phenotypes after inoculation of plants with pathogens (summarized in Bartoli and Roux, 2017 and Xiao *et al.*, 2017). In the context of the microbiome, and in line with most of the research focusing on taxonomic composition, Horton *et al.* (2014) used operational taxonomic unit (OTU) abundance in leaves of 196 Arabidopsis accessions grown in a common garden to identify genes responsible for controlling leaf microbiome composition. Indeed, several significant single nucleotide polymorphism (SNP) associations were found (Horton *et al.*, 2014). They mapped into regions with genes responsible for cell wall synthesis, defence response, and kinase activity; however, no specific genes have been functionally characterized. The function of the candidate genes, however, agrees well with confirmed effects of these processes on the microbiome composition (Bulgarelli *et al.*, 2012; Lebeis *et al.*, 2015) and also with human microbiome studies that also often find association of microbiome composition with immunity (Hall *et al.*, 2017). In a similar approach with 300 accessions from the extended Goodman Maize Association Panel (Flint-Garcia *et al.*, 2005), Wallace *et al*. (2018) showed that only two OTUs and three higher order taxa from leaf microbiomes demonstrated significant heritability. Using the OTUs to infer metagenome content yielded a further 222 heritable traits, represented by predicted functional gene annotations (Wallace *et al.*, 2018). Less than 25% of the features produced significant associations, and the corresponding quantitative trait loci (QTLs) were mostly of a small effect and in the vicinity of genes with unknown function (Wallace *et al.*, 2018).

The experiment using a panel of 196 Arabidopsis accessions has been sampled for composition of root microbiome and analysed in the same way as in Horton *et al.* (2014). Interestingly, this study revealed that the host exerts a larger effect on fungal communities than on bacterial communities (Bergelson *et al.*, 2019). Candidate genes potentially affecting the composition of root microbiome identified in a GWAS were annotated as related to cell wall integrity and immunity, similar to leaves, but also to root and vasculature development (Bergelson *et al.*, 2019).

Only a few studies so far have addressed the effect of plant genetic variation on the function of the microbiome beyond its taxonomic composition. In a pioneering study, using metatranscriptomics, Turner *et al.* (2013) revealed vast differences in abundance and function of microorganisms in the rhizosphere of three different crop species. Most importantly, the rhizospheres of some species were specifically enriched for different metabolic capabilities, such as cellulose degradation in cereals or hydrogen oxidation in pea (Turner *et al.*, 2013). Similarly, rhizoplane bacterial communities of wheat and cucumber grown in the same soil were enriched in expression of genes for nitrate and sulfate reduction, respectively (Ofek-Lalzar *et al.*, 2014). However, the wide phylogenetic distances between the studied species probably prevents any further attempt at identifying the causative genes underpinning the observed microbial diversification. This has been possible in a study by Koprivova *et al.* (2019), who measured microbial aryl-sulfatase activity in soils after cultivation of Arabidopsis accessions. While several studies showed only a small impact of the accessions on microbiome structure (Bulgarelli *et al.*, 2012), this analysis revealed up to a 10-fold difference of sulfatase activity in soil from different genotypes (Koprivova *et al.*, 2019), showing the importance of focusing on functional rather than taxonomic traits. Furthermore, the microbial sulfatase activities were used for GWAS to identify Arabidopsis genes affecting the microbial community of the rhizosphere. The candidate genes included genes involved in sulfur metabolism and, as expected, in secondary metabolism. Among them one candidate has been analysed in detail to show that the phytoalexin camalexin, which is an important component of plant immunity against leaf fungal pathogens, has a role in plant–microbe interactions in the rhizosphere (Koprivova *et al.*, 2019). In the functionality context, the approach of Wallace *et al.* (2018) to predict microbiome function from host genetics has strong potential to formulate working hypotheses, and it would be interesting to see it applied more widely and to compare derived predictions against the measured metabolic functions of the microbial community.

One agriculturally relevant function of soil microbiota is the plant growth promotion (PGP) effect, which can be caused by different mechanisms. Haney *et al.* (2015) found that Arabidopsis accessions varied in their ability to host the root-associated bacterium *Pseudomonas fluorescens* WCS365, and also in the ability to gain from the PGP properties of other *Pseudomonas* strains. The variation in growth promotion was also associated with variation in protection against the root pathogen *Fusarium oxysporum* (Haney *et al.*, 2015). In a more detailed study, Wintermans *et al.* (2016) compared the extent to which Arabidopsis accessions profited from incubations with the PGP rhizobacterium *Pseudomonas simiae* WCS417r. The accessions showed large variation in fresh weight gain, proliferation of lateral roots, and elongation of the primary root upon exposure to the bacterium (Wintermans *et al.*, 2016). GWAS analysis yielded several highly significant associations, and consequently candidate genes potentially influencing the susceptibility of Arabidopsis to the PGP effects of this strain; unfortunately, these were not further tested (Wintermans *et al.*, 2016). However, these analyses together with the multiple GWAS on plant–pathogen interaction and on human microbiome show very clearly that the exploitation of natural variation is a powerful approach to identify the mechanisms by which plants shape their microbiomes.

## Plant secondary metabolites shown to affect the microbiome

Several recent studies have shown that secondary metabolites play a distinct role in fine-tuning the composition and function of the rhizosphere microbiome (Table 2) (Lebeis *et al.*, 2015; Stringlis *et al.*, 2018; Cotton *et al.*, 2019; Koprivova *et al.*, 2019; Voges *et al.*, 2019). Using sensitive analytical techniques, untargeted metabolomic profiling has revealed that root tissues and exudates contain hundreds of secondary metabolites from diverse molecular classes (Neal *et al.*, 2012*a*; Mönchgesang *et al.*, 2016; Hu *et al.*, 2018; Yuan *et al.*, 2018; Zhalnina *et al.*, 2018). Mechanistically, plant secondary metabolites can exert a wide spectrum of effects upon individual microbial strains, by functioning as signalling molecules, nutrient sources, or as toxins. This provides a wide scope for secondary metabolites to act as causative agents shaping the biochemical ecology of the rhizosphere (Jacoby *et al.*, 2020; Pascale *et al.*, 2020). Indeed, a number of secondary metabolites have recently been unequivocally shown to affect microbiome composition and/or function. They belong to different chemical and functional classes and, therefore, they presumably have different mechanisms of action on the communities. Compounds involved in plant immunity and response to pathogens seem, however, to be the most common group of the metabolites active in the rhizosphere.

**Table 2.** Summary of recent literature giving new insights into the specific secondary metabolites shaping the root microbiome

Secondary metabolite	Effects on microbiome	Growth system	Functional mechanism	Target microorganism	Reference
Coumarins	• Altered microbial community assembly	• Arabidopsis; pots	• Differential microbial toxicity	• *Verticillium dahliae* JR2 • *Fusarium oxysporum* f. sp. *raphani*	• Stringlis *et al.* (2018)
	• Altered SynCom assembly	• Arabidopsis; hydroponics	• ROS production	• *Pseudomonas* sp. Root329	• Voges *et al.* (2019)
Benzoxazinoids	• Altered microbial community assembly • Soil legacy of pathogen suppression	• Maize; field and pots	• Plant–soil feedback	• OTUs belonging to Proteobacteria and Chloroflexi	• Hu *et al.* (2018)
	• Altered microbial community assembly	• Maize; pots	• Metabolic regulation	• OTUs belonging to Methylophilaceae and Xanthomonadaceae	• Cotton *et al.* (2019)
	• Altered microbial community assembly	• Maize; pots	• Gatekeeper effects	• OTUs belonging to Proteobacteria and Chloroflexi	• Kudjordjie *et al.* (2019)
Camalexin	• Altered associations with plant growth-promoting bacteria	• Arabidopsis; plates	• Differential microbial toxicity	• *Pseudomonas* sp. CH267	• Koprivova *et al.* (2019)
Glucosinolates	• Altered association with a plant growth-promoting fungus	• Arabidopsis; plates	• Integration of immune and nutrition status	• *Colletotrichum tofieldiae*	• Hiruma *et al.* (2016)
	• Restriction of excessive fungal proliferation	• Arabidopsis; plates	• Toxicity of glucosinolate breakdown products	• *Serendipita indica* • *Sebacina vermifera*	• Lahrmann *et al.* (2015)
Triterpenes	• Altered microbial community assembly	• Arabidopsis; pots	• Differential impact on microbial growth rates	• *Arthrobacter* sp. strain A224 • *Agromyces* sp. strain A475-1	• Huang *et al.* (2019)

### Glucosinolates

The glucosinolates are one of the best studied classes of defence compounds found in the Brassicaceae (Halkier and Gershenzon, 2006). These sulfur-containing metabolites were originally described as defence against herbivores, as after tissue damage they are metabolized by myrosinase into toxic and deterrent isothiocyanates, nitriles, or other products. However, they are also part of antifungal and antibacterial machinery and are found in root exudates, all prerequisites for compounds shaping the microbiome (Bednarek *et al.*, 2009; Mönchgesang *et al.*, 2016). It has been long known that brassica plants affect soil microbiota and it was exploited for disease control (Papavizas, 1966). These effect were attributed to the degradation products of glucosinolates, for example showing a correlation between the concentration of phenylethylisothiocyanate in the rhizosphere and bacterial community structures (Rumberger and Marschner, 2003). Similarly, engineering Arabidopsis to produce *p*-hydroxybenzylglucosinolate has resulted in significant changes in the microbial community (Bressan *et al.*, 2009). Glucosinolates also have an impact on association of Arabidopsis with endophytic fungi. Indolic glucosinolates, derived from tryptophan, accumulate upon inoculation with *Serendipita indica* or *Sebacina vermifera*, which naturally colonize Arabidopsis roots (Lahrmann *et al.*, 2015). Mutants in indolic glucosinolate synthesis show a highly increased colonization by the fungi, pointing to their important role in maintaining the mutualistic interaction with these fungi (Lahrmann *et al.*, 2015). Also *Colletotrichum tofieldiae*, another naturally occurring colonizer of Arabidopsis, requires indolic glucosinolates for exerting its PGP effect, and at least some indolic phytoalexins to prevent it from turning into a pathogen (Hiruma *et al.*, 2016). Glucosinolate patterns of Arabidopsis accessions are highly diverse, also affecting the root exudates (Mönchgesang *et al.*, 2016); therefore, glucosinolates are good candidates for metabolic signals to drive the composition of the microbiomes. This has, however, not been apparent in the available GWAS data so far.

### Camalexin

On the other hand, the importance of camalexin, another sulfur-containing indolic phytoalexin, for shaping the root microbiome was revealed first by GWAS. Camalexin, 3-thiazol-2'-yl-indole, derived from tryptophan, is one of the major phytoalexins of Arabidopsis (Glawischnig, 2007). Camalexin plays an important role in the response to the necrotrophic pathogens *Alternaria brassicicola* and *Botrytis cinerea*, and the oomycete *Phytophthora brassicae* (Thomma *et al.*, 1999; Rowe and Kliebenstein, 2008; Schlaeppi *et al.*, 2010). Most of the research on camalexin focused on its role in pathogen defence in the leaves. However, camalexin was also shown to be exuded from roots upon elicitation with flagellin (Millet *et al.*, 2010) and to affect several root-specific plant–microbe interaction-related traits (Koprivova *et al.*, 2019). A gene for a new isoform of cytochrome P450 was found in a GWAS screen for variation in microbial sulfatase activity in the rhizosphere soil. Loss of function of this gene, and other genes for camalexin synthesis, was associated with lower sulfatase activity and lower accumulation of camalexin in roots of soil-grown plants. Both phenotypes could be complemented by exogenous camalexin, providing evidence for its function in the soil (Koprivova *et al.*, 2019). In addition, these camalexin-deficient Arabidopsis mutants were unable to benefit from PGP effects, which several mutualistic bacterial strains confer on the wild-type plants, shown by increased biomass. While the direct effect of camalexin on the microbial community structure remains to be shown, the loss of all indolic secondary metabolites in the mutant with disrupted function of both CYP79B2 and CYP79B3 affected the abundance of individual strains in a SynCom (Voges *et al.*, 2019).

### Benzoxazinoids

Glucosinolates and camalexin are prominent examples of metabolites affecting microbiome assembly, because they are synthesized by the model plant Arabidopsis; however, they are specific to Brassicaceae. Another group of secondary metabolites that has been shown to shape the soil microbiota are the benzoxazinoids (de Bruijn *et al.*, 2018). Benzoxazinoids are indole-derived compounds found mainly in the grasses and crops, such as maize, where they are important for insect resistance (Niemeyer, 2009). They are also exuded from the roots and serve as allelochemicals or protectants against pathogens, similar to camalexin or glucosinolates (de Bruijn *et al.*, 2018). However, at the same time, they can also act as chemoattractants for PGP bacteria in the rhizosphere (Neal *et al.*, 2012*b*). It is therefore not surprising that benzoxazinoids affect the composition of the root microbiome (Hu *et al.*, 2018; Cotton *et al.*, 2019; Kudjordjie *et al.*, 2019). These three reports employed maize mutants in benzoxazinoid synthesis to reveal alteration in both bacterial and fungal communities. Interestingly, Hu *et al.* (2018) was able to link the shifts in microbiota with pathogen resistance of the next plant generation, revealing benzoxazinoids to be part of the plant–soil feedback mechanism, at least for cereals.

### Coumarins

Another group of secondary metabolites involved in shaping the root microbiome are ubiquitous in the plant kingdom. Coumarins were first studied in the context of the human microbiome, because many health-promoting natural products as well as toxins belong to this class of metabolites. For example, feeding rats ochratoxin A, one of the major mycotoxins derived from a coumarin backbone, resulted in increased abundance of a *Lactobacillus* strain that is able to absorb this metabolite (Guo *et al.*, 2014). In plant roots, secreted coumarins have been recognized as important for increasing bioavailability of iron, through reduction and chelation (Tsai and Schmidt, 2017), and also for their antimicrobial functions (Beyer *et al.*, 2019; Stringlis *et al.*, 2019). Interestingly, a transcription factor, MYB72, which controls expression of several genes in coumarin synthesis, is also important for induced systemic resistance against pathogens triggered by the root bacterium *Pseudomonas simiae* WCS417 (Zamioudis *et al.*, 2014). However, exudation of coumarins also has an impact on the microbial communities, as shown by analyses of microbiota assemblies of Arabidopsis *myb72* and *f6′h1* mutants that do not secrete scopoletin (Stringlis *et al.*, 2018; Voges *et al.*, 2019). As predicted from the function of coumarins in iron mobilization, the effects on the communities were apparent in iron-deficient soils, and were shown to inhibit a selection of pathogenic fungi, whereas a number of beneficial PGP rhizobacteria were tolerant (Stringlis *et al.*, 2018). There is some evidence that a scopoletin-tolerant *Pseudomonas* strain can stimulate iron uptake in Arabidopsis (Verbon *et al.*, 2019), so a promising target for future research could involve boosting plant iron nutrition via coumarin-mediated shaping of the root microbial community.

### Triterpenes

Triterpenes are an abundant and ubiquitous class of secondary metabolites with known antimicrobial effects (Papadopoulou *et al.*, 1999). A large variety of triterpenes are produced in Arabidopsis roots and some of their biosynthetic genes are induced by jasmonate, pointing to a possible role in plant–microbe interactions (Huang *et al.*, 2019). Indeed, mutants in key genes of triterpene synthesis, affecting thalianin, arabidin, and several triterpene fatty acid esters, showed distinct alterations in microbiome assembly when grown in natural soil (Huang *et al.*, 2019). Interestingly, the OTUs affected by disruption of triterpene synthesis were enriched in bacterial OTUs specific for Arabidopsis. It is thus possible that plant species-specific triterpenes are instrumental in selection of species-specific strains for root microbiome assembly. To test this hypothesis more plant species need to be analysed.

## Conclusion and perspectives

One of the major questions in the field of plant–microbe interactions involves defining the functional mechanisms that plants use to shape their microbiome. This is a strategic priority, because these traits could be targeted in crop breeding programmes to develop more sustainable agriculture. Secondary metabolites have often been framed as a plausible mechanism for fine-tuning the plant microbiome, because these chemically diverse molecules frequently exhibit bioactive properties against microbes, and the extensive variation in metabolite profiles between plant species could explain some of the interspecies differences in microbial community assembly (Fitzpatrick *et al.*, 2018). However, until recently, there was a lack of empirical evidence defining the specific plant metabolites that modulate the root microbiome.

There is now a rapidly expanding body of literature unequivocally showing that plant secondary metabolites affect microbiome composition and function (Table 2). Methodologically, these recent studies have synthesized plant genetics with microbiological techniques, often by analysing the altered microbial communities that assemble on the roots of mutant plants impaired in the biosynthesis of a specific secondary metabolite. Increasingly, synthetic community approaches are being used to define the individual microbial strains that are enriched or depleted according to specific plant metabolites. Although synthetic communities will always under-represent the true diversity of a natural microbiome, their key advantage is the ability to address specific questions. For instance, if one individual strain exhibits a particularly strong enrichment or depletion according to plant genotypes differing in secondary metabolite profiles, then this strain can be studied in follow-up experiments, to define the biochemical mode of action exerted by the metabolite on the microorganism, such as reactive oxygen species-mediated toxicity (Voges *et al.*, 2019).

Over the next few years, we anticipate that further studies will continue to advance the mechanistic understanding of how secondary metabolites affect the microbiome. The existing literature has only scratched the surface of plant metabolic diversity, particularly because the majority of them are focused on Arabidopsis. Therefore, genetic manipulation of other metabolite biosynthesis pathways across diverse plant species is almost certain to generate plants with altered microbial associations. Furthermore, untargeted metabolomics measurements are becoming more widespread and accessible, with new approaches now enabling the integration of root metabolomic profiles with rhizosphere microbiome composition (Cotton *et al.*, 2019).

In the longer term, it is tempting to speculate that secondary metabolites could be used as a tool for tailoring the plant microbiome. For example, breeding programmes could aim to rationally manipulate root chemistry by incorporating the entire biosynthetic pathway required to produce a targeted secondary metabolite, in order to recruit a beneficial strain or to deter a pathogen. To achieve this aim, one option could involve large-scale genome editing to introduce the necessary alleles into cultivated plants. Of course, efforts to move from discovery science to translation will probably encounter obstacles, particularly if the secondary metabolites which have the strongest influence on the microbiome were counter-selected during domestication and breeding due to their negative impact on palatability. Despite this, we still feel that secondary metabolites represent promising targets for rationally manipulating the plant microbiome, particularly because one of the selection pressures which favoured the evolution of these bioactive molecules was probably their capacity to influence the composition and function of the root microbial community.
